# A remarkable assemblage of petroglyphs and dinosaur footprints in Northeast Brazil

**DOI:** 10.1038/s41598-024-56479-3

**Published:** 2024-03-19

**Authors:** Leonardo P. Troiano, Heloísa B. dos Santos, Tito Aureliano, Aline M. Ghilardi

**Affiliations:** 1 The National Historic and Artistic Heritage Institute, Brasília, DF 70390-135 Brazil; 2 Institute of Cariri Archaeology Dr. Rosiane Limaverde, Casa Grande Foundation, Nova Olinda, CE 63165-000 Brazil; 3 Department of Zoology, Regional University of Cariri (URCA), Crato, CE 63105-000 Brazil; 4 Diversity, Ichnology and Osteohistology Laboratory (DINOLab), Department of Geology, Federal University of Rio Grande do Norte (UFRN), Natal, RN 59075-000 Brazil

**Keywords:** Archaeology, Palaeontology

## Abstract

The Serrote do Letreiro Site, found on the northwest periphery of the Sousa Basin, Brazil, presents a remarkable convergence of paleontological and archaeological elements. It is constituted of sub-horizontal "lajeiros", or rock outcrops, intermingled with endemic Caatinga vegetation. The three prominent outcrops feature fossilized footprints of theropod, sauropod, and iguanodontian dinosaurs from the Early Cretaceous Period. Adjacent to these dinosaur tracks, indigenous petroglyphs adorn the surface. The petroglyphs, mainly characterized by circular motifs, maintain a striking resemblance to other petroglyphs found in the states of Paraíba and Rio Grande do Norte. This study primarily endeavors to delineate the site's major characteristics while concentrating on the relationship between the dinosaur footprints and the petroglyphs. It concurrently assesses the preservation status of this invaluable record, shedding light on its implications for the realms of paleontology, archaeology, and cultural heritage studies.

## Introduction

The “Serrote do Letreiro” Site, tentatively translated here as “Signpost Hill”, is located in the Sousa municipality, Paraíba State, Brazil, and is characterized by an outstanding juxtaposition of paleontological and archaeological elements (Fig. 2). The site comprises three large rock outcrops, totaling more than 15,000 square meters in size. The geological context is attributed to the Antenor Navarro Formation (Sousa Basin), Berriasian-Hauterivian in age (Early Cretaceous), predominantly consisting of conglomerate sandstone, characteristic of an alluvial fan paleoenvironment. Within the paleontological interest, the site presents footprints of theropod, sauropod, and ornithopod dinosaurs^1^. Of archaeological interest are numerous low-relief petroglyphs mainly consisting of circles filled with radial lines and other motifs of deferred recognition, meaning, not easily recognizable, tending to be considered geometric (see Magalhães^2^, p.195).

**Figure 1:**
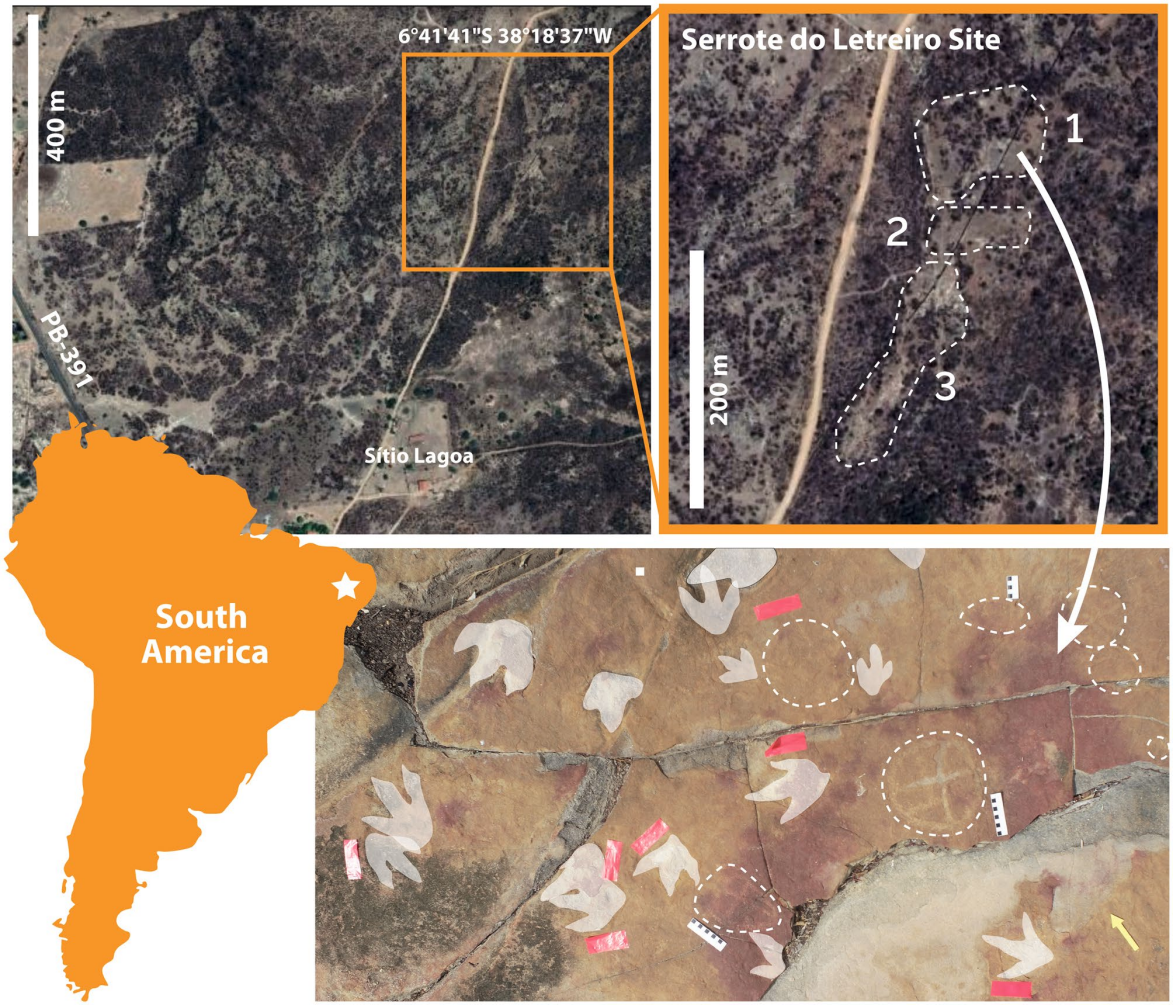
Location of the “Serrote do Letreiro” Site and central area of the first and northernmost outcrop, with a high concentration of footprints and petroglyphs. (1) Outcrop one, also referred to as Northermost outcrop; (2) Outcrop two, also referred to as middle/central outcrop; (3) Outcrop three, also referred to as Southernmost outcrop. Footprints in highlight and petroglyphs circled by dashed lines. Coordinates are given in the WGS 84 datum. Satellite photography from Google Earth version 7.3 © Google, Airbus, Maxar Technologies. Map generated with Adobe Illustrator 2019.

The site is located 11 kilometers from the urban center of Sousa Municipality, in the Sertão Paraibano region, State of Paraíba, Northeast Brazil. It is situated in the rural property Sítio Lagoa, accessible via Highway PB-391 (Fig. 1). The site is within the broader context of the Vale dos Dinossauros Natural Monument (Known as Dinosaur Valley), an important natural site located in the Sousa Basin, characterized by the presence of numerous ichnofossils, all dating back to the Early Cretaceous Period^3^. Access to Serrote do Letreiro is via a dirt road, but direct access involves overcoming obstacles presented by the local xerophilic Caatinga vegetation and barbed-wire fences, installed to prevent cattle from passing through. The climate conditions prevailing at the site are consistent with the typical markers of the northeastern semi-arid region, defined by intense insolation, temperatures reaching values close to 40°C, and prolonged periods of rainfall scarcity.

The first mentions of dinosaur footprints from the Sousa region date back to the early 20th century. However, the paleontological record only became subject to scientific investigation with Giuseppe Leonardi, who began research in the area in 1975, and is credited with the formal discovery of several paleontological sites, including Serrote do Letreiro, found during the 1979 expedition (see pages 102–107 in Leonardi and Carvalho^3^). Leonardi's research concentrated on the paleontological aspects of the site. Regardless, as early as the 1979 publication, the author mentioned the existence of petroglyphs, and referred to them only as "Cariri Indian carvings." Brief mentions reappeared in the years 2001^4^ and 2002, via a volume published by the National Department of Mineral Production (DNPM, now the National Mining Agency). In 2020, the site was listed on the "Geossit" platform of the Geological Survey of Brazil, which registers national geosites. Additionally, the site is classified on the platform as one of international interest due to the unique intersection between archaeological and paleontological components present there. The site was most recently described by Leonardi and Carvalho in 2021^1^, who again focused on its paleontological aspects.

Despite brief mentions, to date no comprehensive analysis of this set of rock art expressions has been carried out, nor have studies taken into account the affinity between the footprints and the petroglyphs. Comparable but not identical associations between fossils and rock art have been described elsewhere. In Australia, for instance, petroglyphs were interpreted as human reproductions of dinosaur footprints by Herbert Basedow^5^ (see pages 195–211). In Poland, a dinosaur footprint was identified at a site of possible occult gatherings^6^. In other sites located in the state of Utah (United States), petroglyphs and fossilized footprints also coexist, even though to a lesser extent. These sites include Poison Spider Dinosaur Tracks, Parowan Gap, and Zion National Park^7^. Regardless, in none of these instances do the petroglyphs display such a close-knit relationship with the footprints as in Serrote do Letreiro, where it is unquestionable that the engravers acknowledged the footprints and intentionally executed the petroglyphs around them, establishing a symbolic connection between human graphic expression and the fossil record. Given the outstanding qualities of this site, this study aims to preliminarily report the discoveries made, as well as to analyze the petroglyphic iconographic program, examining the spatial and unusual connection between them and the dinosaur footprints. Additionally, the site's conservation status is assessed, along with proposed protection and preservation measures and actions that could be implemented to safeguard in situ this record.

## Results

**Figure 2:**
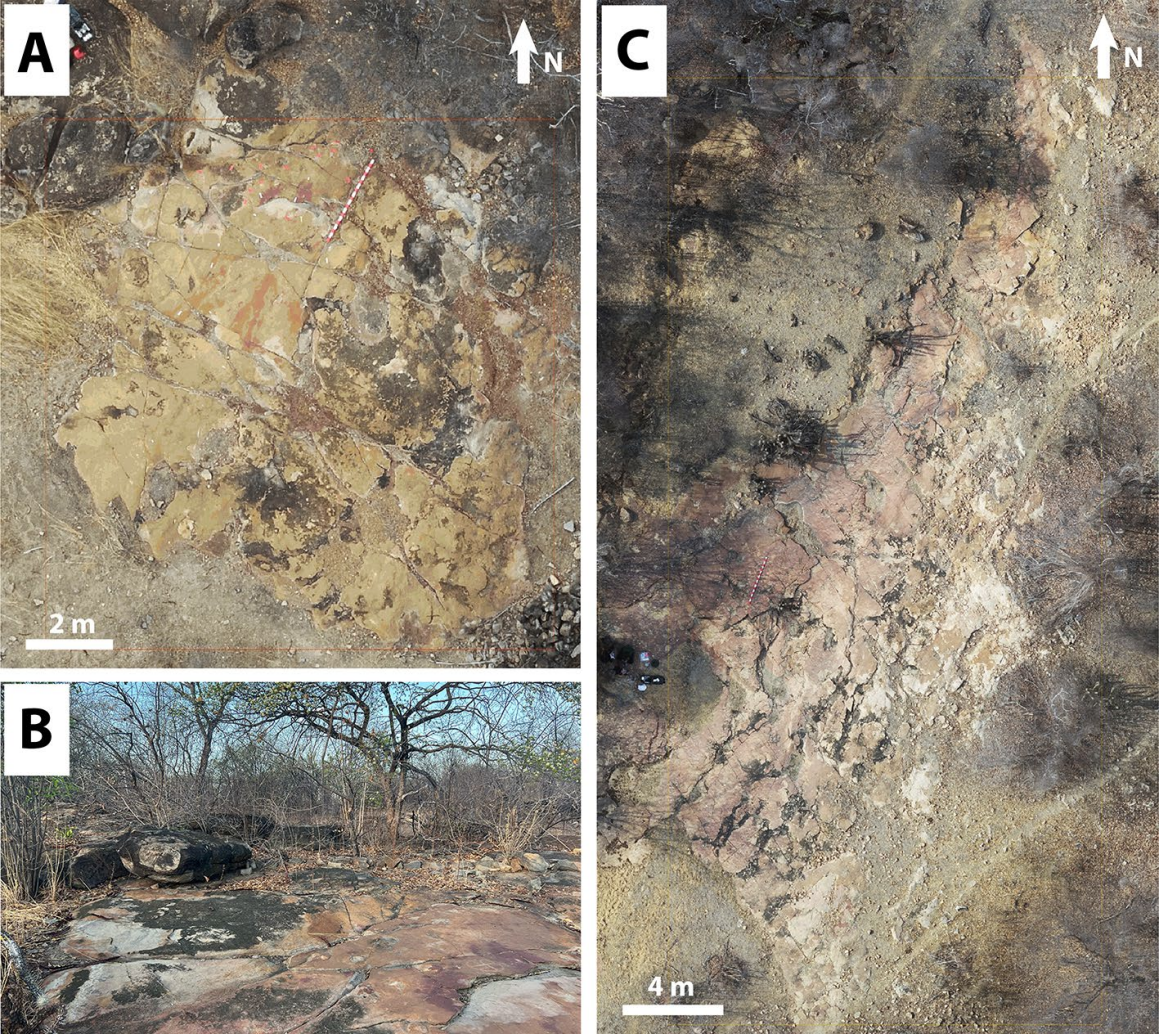
(**A**) Outcrop 1/Northernmost outcrop, scale 1:160. (**B**) Ground-level view of the site from Outcrop 1. (**C**) Outcrop 3/Southernmost outcrop, scale 1:87.

**Figure 3:**
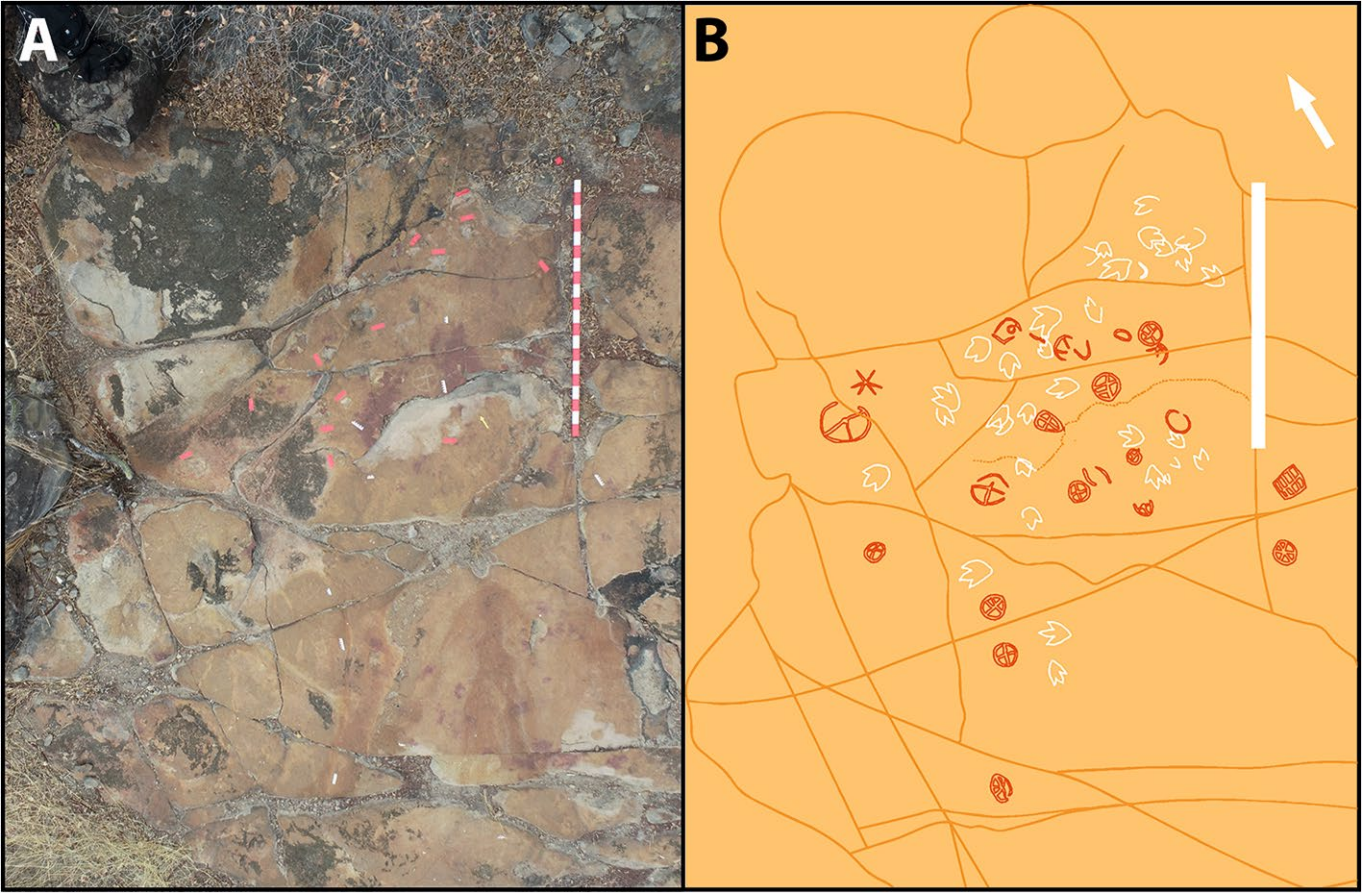
(**A**) Aerial photography of Outcrop 1. (**B**) Digital sketch-map of the same outcrop, highlighting theropod footprints in white and petroglyphs in dark orange. Scale bar = 2 m. The arrow indicates north.

**Figure 4:**
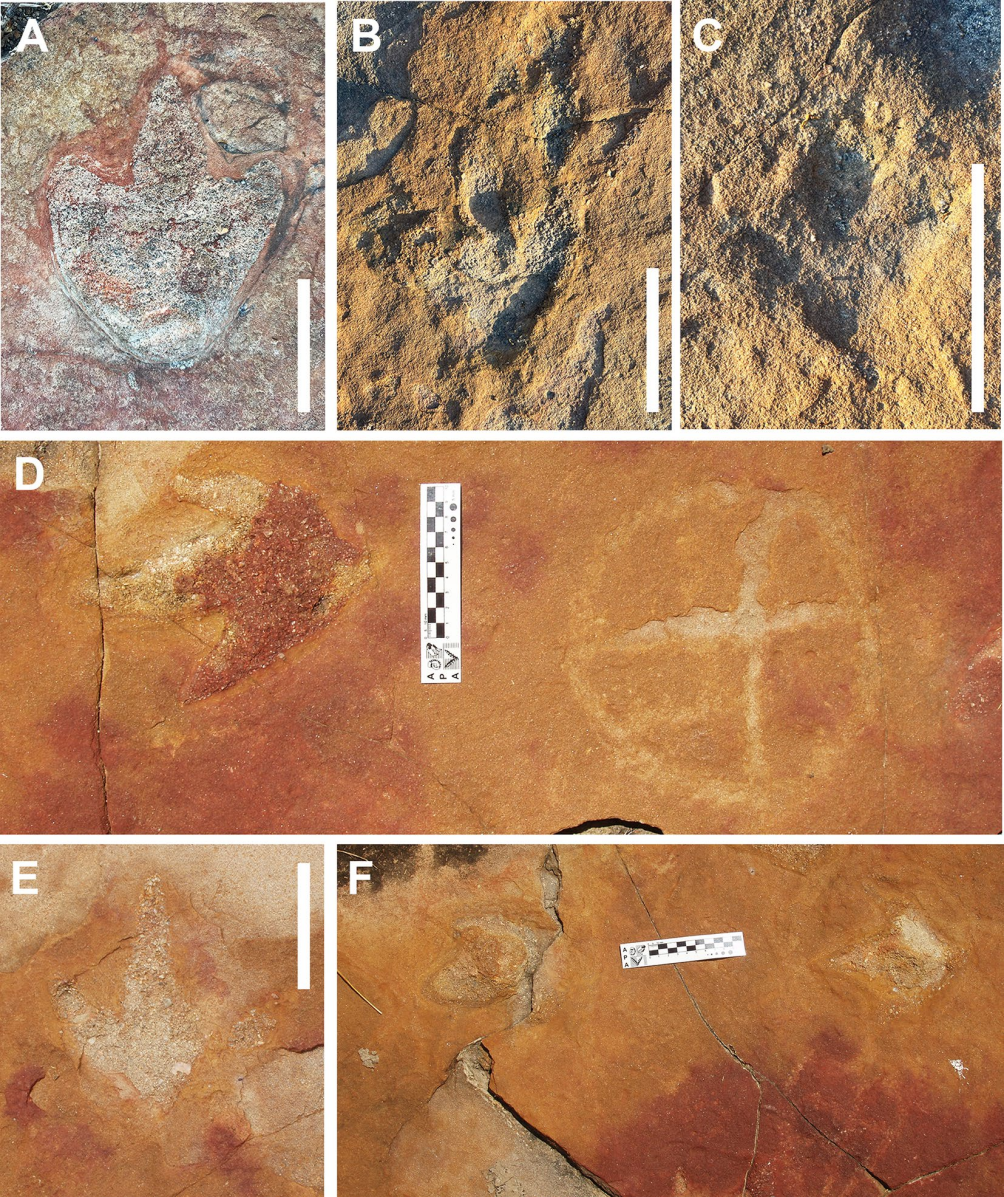
Different morphotypes of tridactyl footprints from outcrop 1, all interpreted as belonging to theropod dinosaurs (**A**–**F**). (**D**) and (**F**) show footprints in close association with petroglyphs. Scale bars = 10 cm.

**Figure 5:**
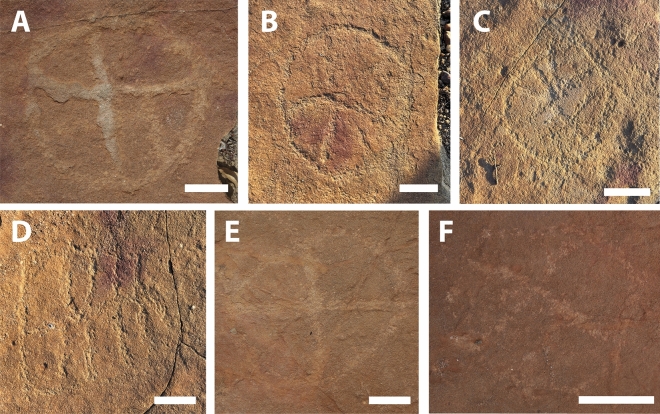
Petroglyphs found at outcrop 1. (**A**–**C**,**E**,**F**) circles internally divided, (**D**) Grid subdivided into eight boxes. Scale bars = 4 cm.

**Figure 6:**
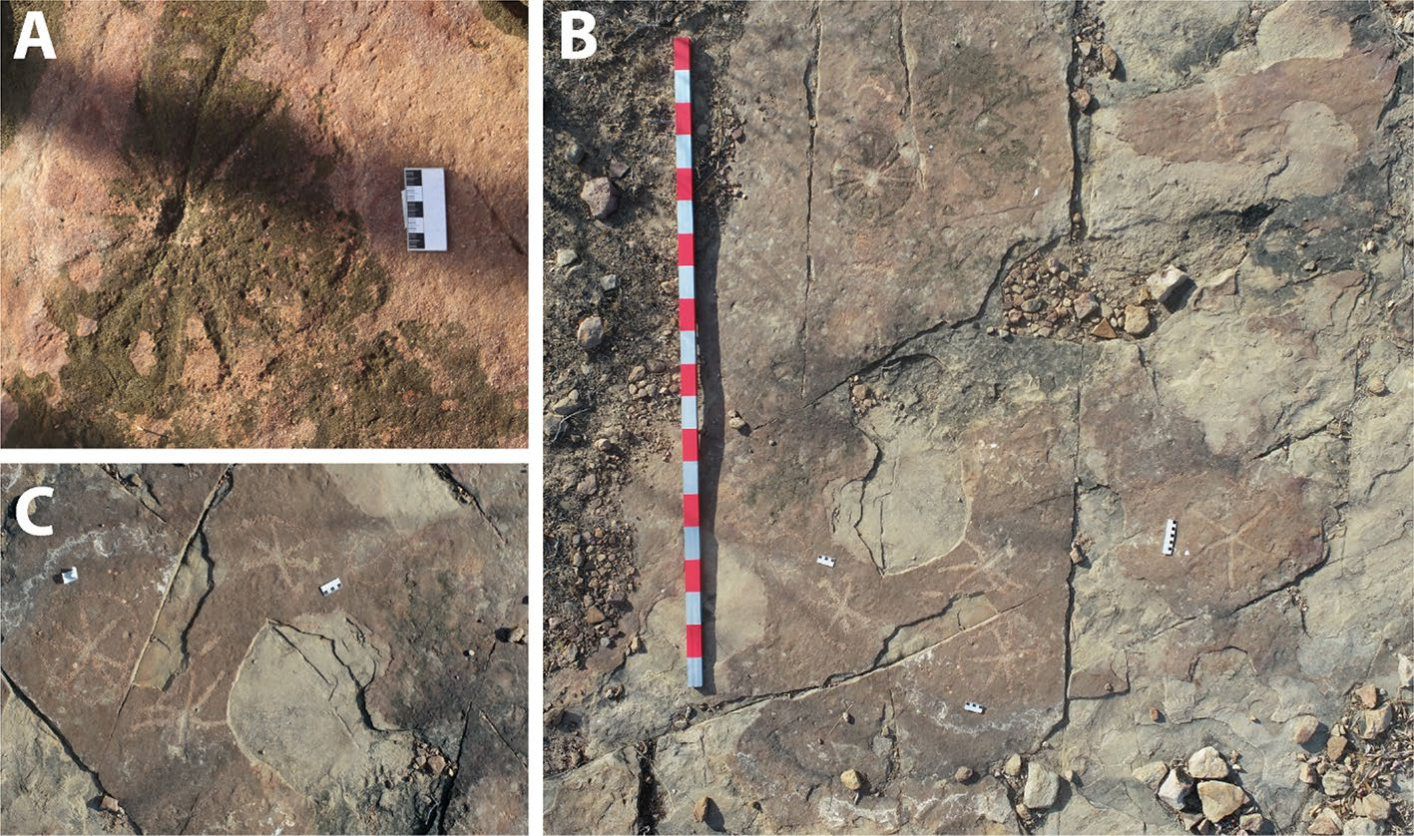
Petroglyphs found at outcrop 3. (**A**) Circle with internal radiating lines; (**B**) Aerial photography in which damage to the graphic horizontal panel is visible, affecting an area of high concentration of petroglyphs; (**C**) Petroglyphs superficially resembling star icons. Most are lines inside a faded circular outline. Scale bars = 5 cm and 2 m.

**Figure 7:**
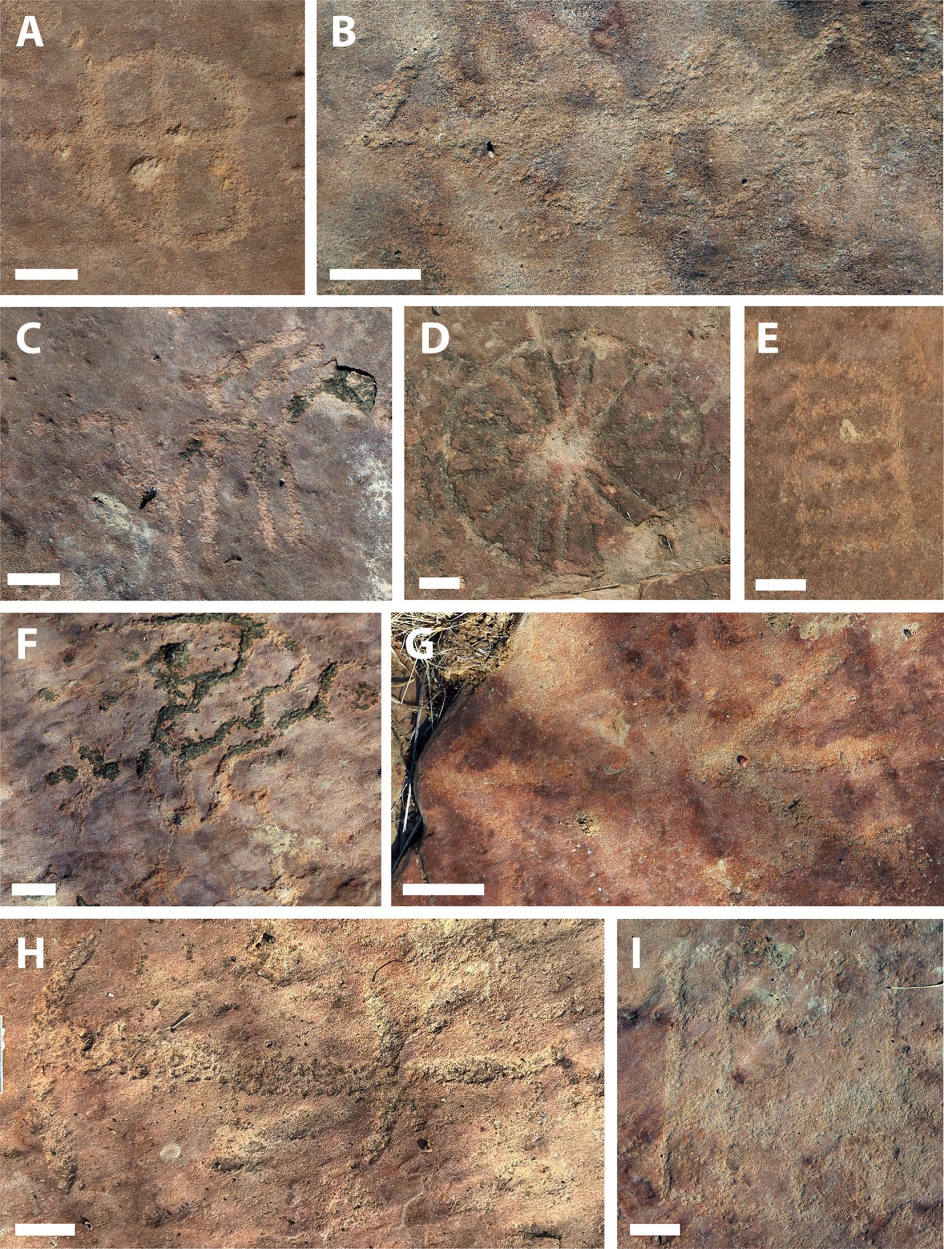
Petroglyphs from the third and southernmost outcrop, consisting of a varied array of motifs. (**A**) Circumference divided into four parts; (**B**) Circles crossed by a central axis; (**C**) Axis with radiating lines, somewhat resembling a phytoform symbol; (**D**) Circle with multiple internal radiations; (**E**) Rectangular form subdivided by four lines; (**F**) Serpentine Motif, grooves filled by moss; (**G**) Tridigits; (**H**) Horizontal line crossed perpendicularly by two small, open curves or half-circles. The employment of the pecking technique is particularly highlighted in this petroglyph; (**I**) Grid divided into sixteen spaces. Scale bars = 4 cm.

**Figure 8:**
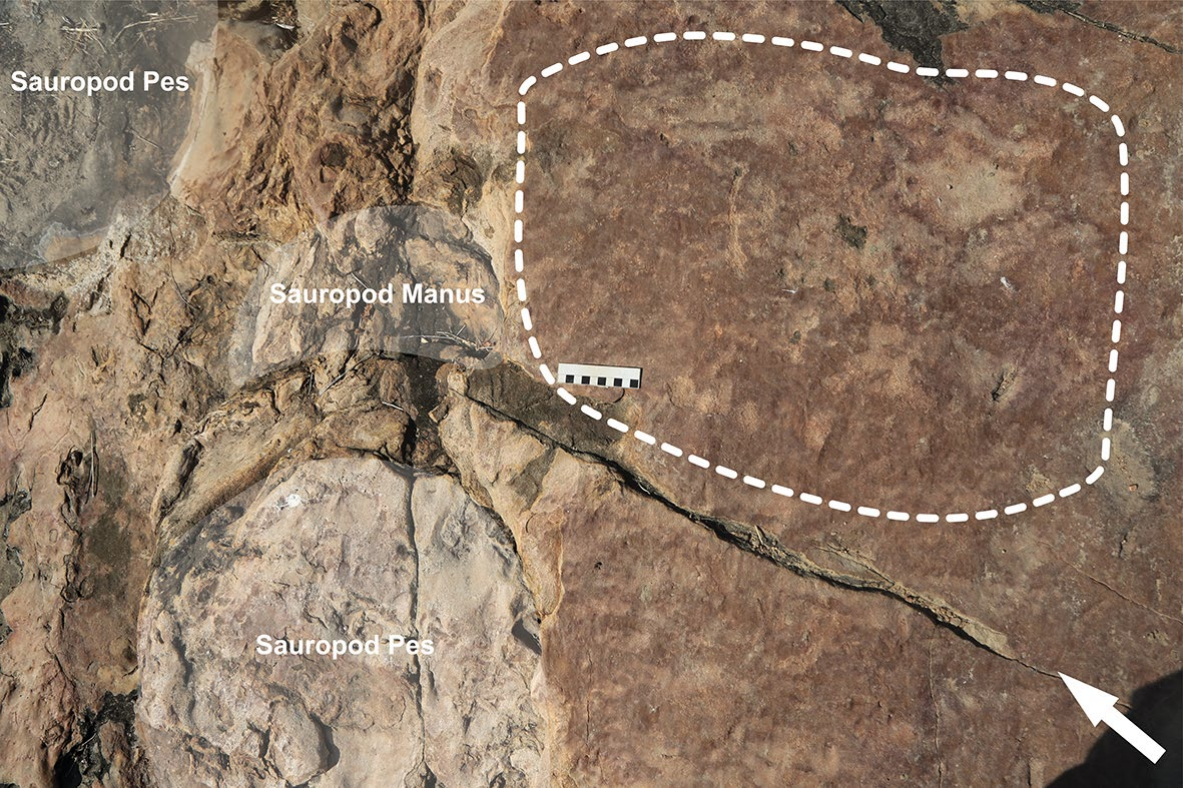
Petroglyphs in close association with sauropod tracks. The dashed line represents the area where there are pecking marks on the rock surface, indicating the presence of petroglyphs. Scale bar = 10 cm.

Interspersed with small pockets of dense local vegetation, three main large outcrops were identified, where both dinosaur footprints and petroglyphs were observed. The petroglyphs were concentrated in two main areas, outcrops 1 and 3 (Figs. 2, 3, 4, 5, 6, 7 and 8). Alongside the petroglyphs briefly mentioned in previous paleontological research, twenty-two complete or minimally recognizable symbols were also identified on outcrop 1, along with the highest concentration of theropod tracks (Fig. 3). At outcrop number 2, only two petroglyphs were located, although current preservation conditions do not allow for a formal understanding of these. During field survey procedures, no mobile objects such as scrapers, lithic artifacts, or other traces of occupation were identified. The survey also located a vast number of markings denoting the existence of an initially much higher number of petroglyphs that have weathered to the point of being no longer legible. At outcrop 3, the southernmost one, thirty petroglyphs and an extensive amount of pecking marks were identified, also suggesting, as in outcrops 1 and 2, that the graphic panel once supported a much larger number of petroglyphs.

The lithological support on which petroglyphs and dinosaur tracks are located are sub-horizontal sandstone and conglomerate sandstone strata situated on a small hill, known as "Serrote”. There is evident displacement of sizable surface portions due to rainwater runoff on the inclined support. The makers’ preference for such type of sandstone is commonly observed in the Northeast region of Brazil, where many of these rock formations were used as support for petroglyphs. Additionally, the site in question is positioned near a water source, specifically a small lake and a temporary stream, which is likewise a recurring factor in similar sites in the region^8^. The process of creating the petroglyphs involved the use of mixed techniques. The petroglyphs were first carved by scraping with light contact between an abrasive instrument and the rock surface. Irregularities are observed on the inner edges of the rock grooves, a result of fast movement, causing friction between the instrument and the surface. Many of the petroglyphs underwent subsequent pecking so that the engraving line was refined by a series of continuous impacts using a sharp instrument. This complementary process resulted in small and repeated concavities of greater depth than those observed in petroglyphs made only through scraping. The preference in some instances for the scraping technique often produces shallow petroglyphs, which renders them more vulnerable to erosion, resulting in the current low visibility and legibility of many petroglyphs.

The petroglyphs in this area are characterized by their deferred recognition nature. They resemble what is now interpreted as geometric forms, but their meaning and whether they were meant to constitute representational or figurative icons remains unknown. Concerning the morphology of the identified petroglyphs, the presence of tetrapartite or pentapartite circumferences stands out. Nevertheless, there are notable exceptions to this trend, including engravings comprised of rectangular grid structures and others resembling stars. Despite the profusion of identified petroglyphs, no overlap was observed between these inscriptions and the fossilized footprints. In none of the cases was it found that the creation of a petroglyph resulted in damage to the existing footprints, suggesting thoughtfulness by the makers. In some cases, there is an extreme proximity between petroglyphs and footprints, with some occurring immediately adjacent to the fossilized tracks (Fig. 4). This only highlights and establishes a more profound relationship between the archaeological and paleontological records. Concerning the graphic identity, or the "set of characteristics that allow attributing a set of graphisms to a particular social authorship"^9^, it was determined that the Serrote do Letreiro site belongs to a broad set of archaeological rock art sites in the Brazilian Northeast region that present an identical repertoire of motifs, either pure or abstract, and of similar or identical execution techniques. In the first rock outcrop, located further north, it is possible to identify a higher concentration of dinosaur tracks. Reports from previous visitors to the site from the past twenty years, as well as statements from residents, indicate that until not long ago, there were a minimum of thirty such petroglyphs that were discernible on outcrop 1. The legible petroglyphs found on this outcrop consist predominantly of circles internally divided by lines (Fig. 5) and are positioned close to the footprints, in some cases as close as a distance of 10 cm.

**Figure 9:**
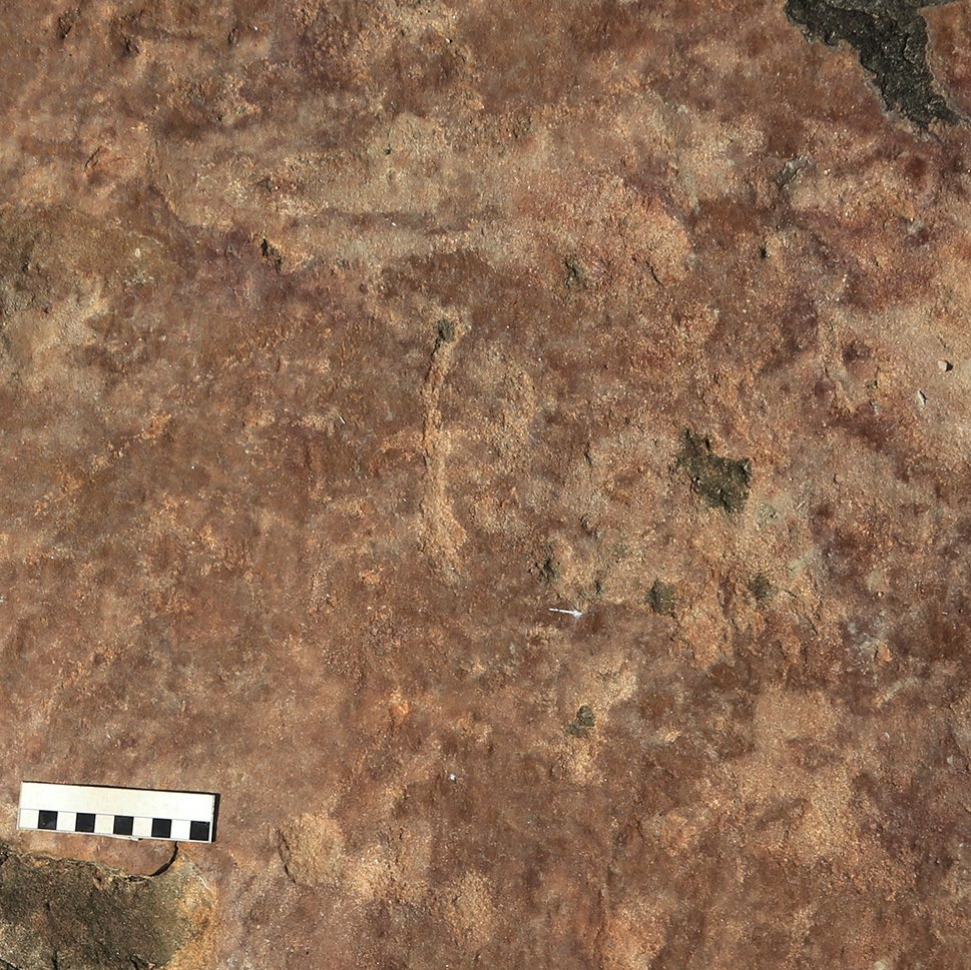
Close-up on the petroglyphs placed near a sauropod track in outcrop 3. Scale bar = 10 cm.

The third outcrop, in the southernmost portion of the site, is of greater extension. This section of the site presents distinct tracks, left mainly by large-size sauropod dinosaurs. This outcrop features a collection of petroglyphs that differ from the inscriptions found in the northern and first outcrop, displaying various motifs (Fig. 7). These include tridigits, grids, several circular shapes with radiating patterns inside the circumference, stars (Fig. 6), and a large motif with serpentine characteristics. In both outcrops where rock art is present, outcrops 1 and 3, the creation process of the petroglyphs involved the application of scraping and pecking techniques. In the third outcrop, petroglyphs are generally not directly associated with the dinosaur footprints. Most are observed in areas where no ichnofossils are present. Exceptions include a few petroglyphs that were placed near a large sauropod trackway (Figs. 8 and 9).

**Figure 10:**
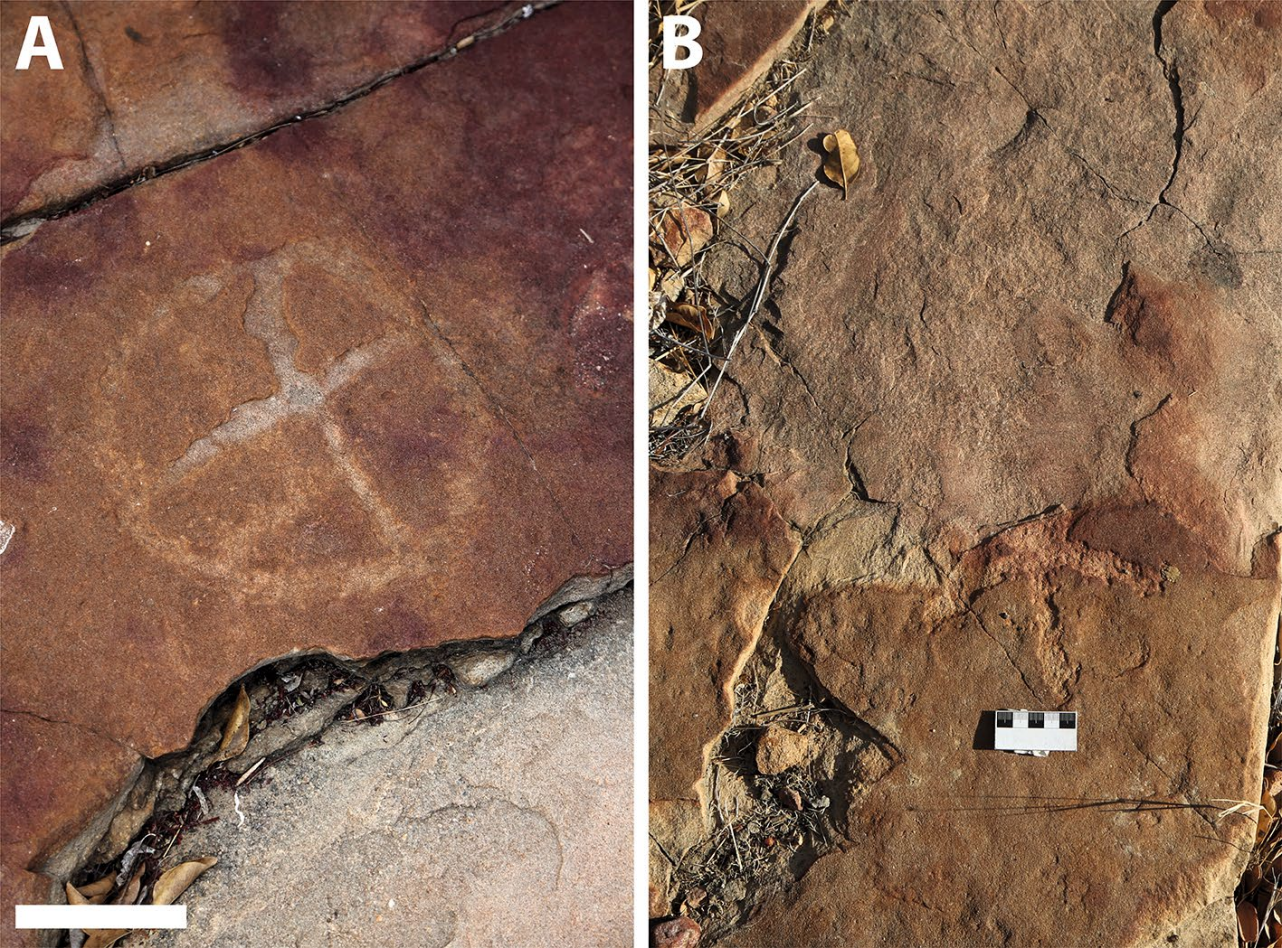
Two out of numerous instances where rock surface spalling has compromised the iconographic program of the site. Scale bar = (**A**) 10 cm and (**B**) 5 cm.

The petroglyphs do not reveal a clear reading direction, and their concentration in the central area of the first, northernmost outcrop, where the theropod footprints specifically occur, is noteworthy. Notably, the authorship of the petroglyphs can be attributed to various individuals. This is supported by the reasonable variation in style observed. Within the realm of graphic expression, style is to be defined as the unique combination of visual elements and techniques utilized by the engraver, which, according to Fernie (See 1995, p. 361, also Sanz & Fiore, 141–159)^10,11^ permits the grouping of works into related categories. These include scenography or placement of symbols, groove depth and thickness, execution method, and size. More specifically, the petroglyphs from the third, southernmost outcrop are predominantly larger, made with deeper incisions into the rock, and form a wealthier repertoire of motifs when compared to the northern outcrop. The authors determine the site's conservation status as precarious, demanding immediate mitigation measures to prevent further damage. The nature of the outcrops, presented in slabs, poses challenges for preserving the archaeological and paleontological records. Instances reveal that spalling or flaking of the layers, often termed the "onion skin effect," has unfortunately disrupted the iconographic program, resulting in severe damage to several petroglyphs and footprints (Fig. 10). Additionally, recent animal scratch marks on the rock surface were noted at the site, alongside significant litter resulting from improper disposal or occasional vandalism by visitors.

## Discussion

**Figure 11:**
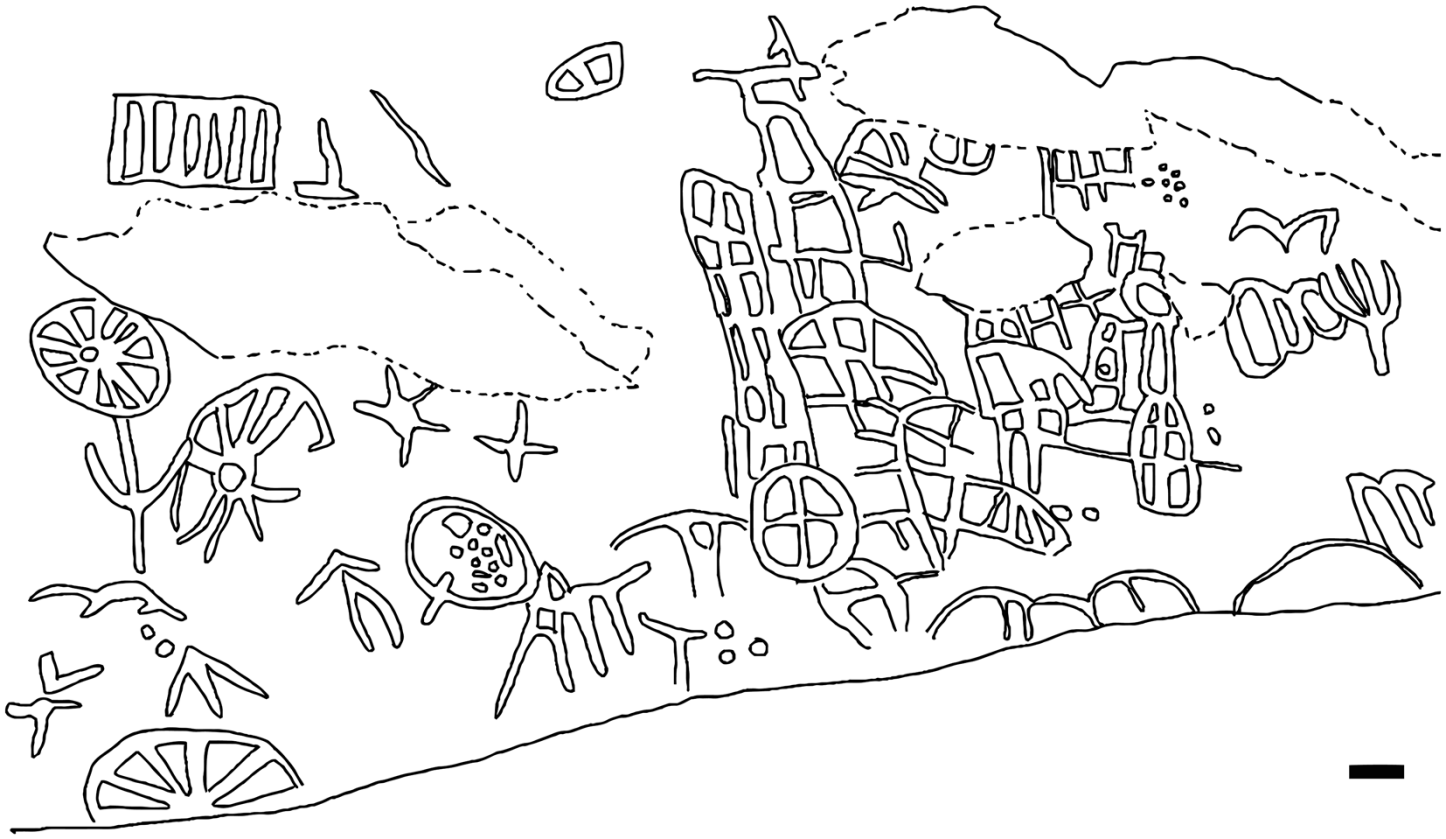
Junco archaeological site, Antônio Martins, Rio Grande do Norte State. The site contains similar and at times identical motifs to the ones found at Serrote do Letreiro, with the same execution techniques. Scale bar = 10 cm. After Santos Júnior^12^.

**Figure 12:**
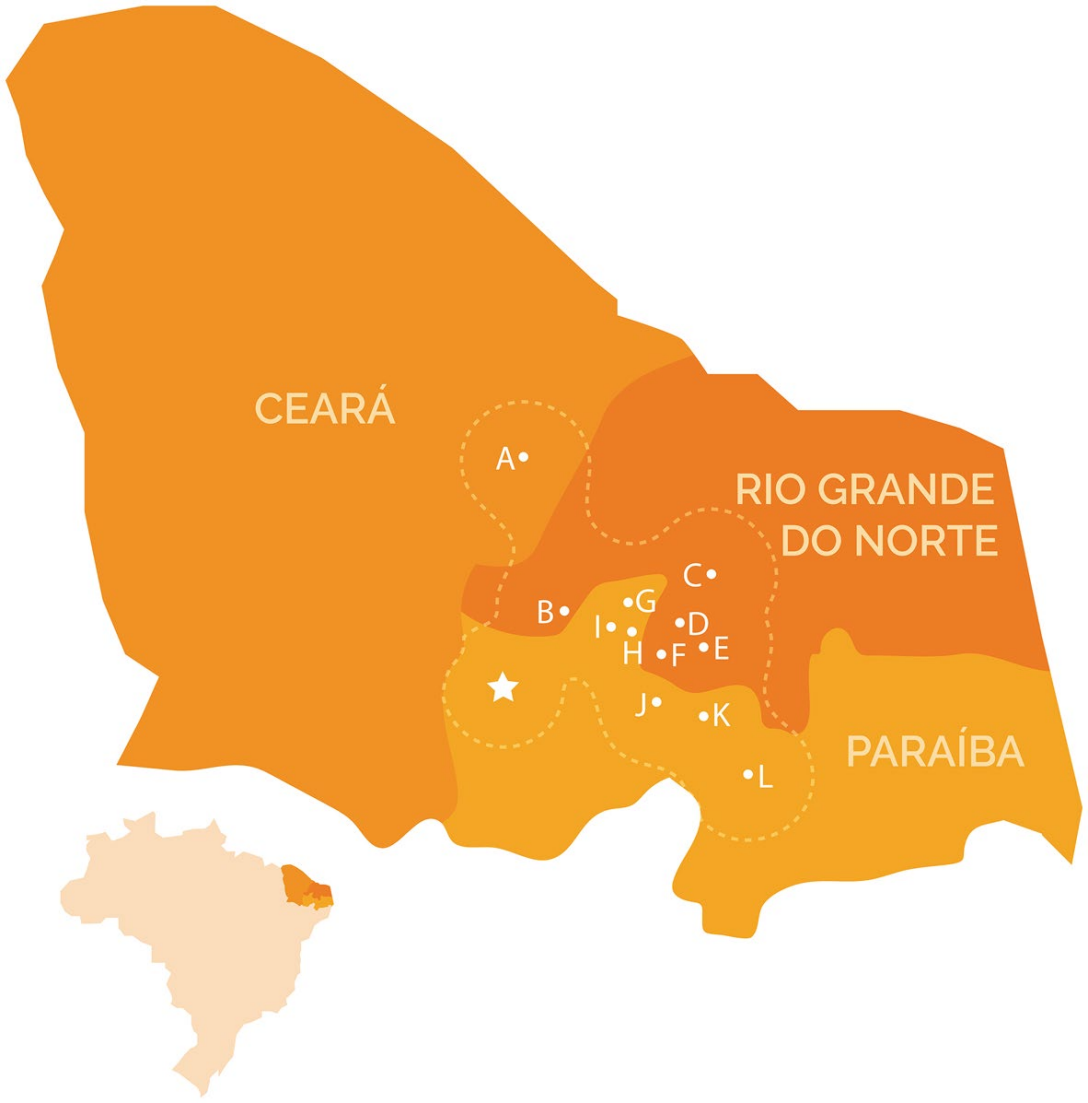
Map indicating cities where archaeological sites with similar or identical petroglyphs to the ones found at Serrote do Letreiro are present. The map covers the states of Ceará, Paraíba, and Rio Grande do Norte, northeast Brazil: (**A**) Alto Santo; (**B**) Antônio Martins; (**C**) Jucurutu; (**D**) Timbaúba; (**E**) Caicó; (**F**) Serra Negra; (**G**) Belém do Brejo Cruz; (**H**) São José do Brejo do Cruz; (**I**) Catolé do Rocha; (**J**) São José de Espinhares; (**K**) São Memede; (**L**) Taperoá. The star represents the Serrote do Letreiro site. The dashed line represents a tentative and hypothetical territory occupied by the native population that could be responsible for the graphic set of petroglyphs in question, based on the distribution of the sites.

The petroglyphs represent a unique and significant record, given their direct association with dinosaur fossil tracks. This ensemble of archaeological and paleontological evidence unequivocally indicates that human populations during the pre-colonial period interacted with and likely assimilated the fossil record, incorporating such record into their graphical expression, a cultural one, and consequently integrating it into its collective identity. Particularly noteworthy is the evident intentionality in creating petroglyphs near the footprints, revealing active engagement with the fossil material, suggesting that these traces not only caught the attention of the native community but were meaningful and became integrated into their knowledge repertoire. In the context of the social authorship of these petroglyphs, they are to be attributed to a human group that once occupied the territory corresponding to the present-day states of Paraíba and Rio Grande do Norte during the pre-colonial period. Substantial evidence for this, especially similar petroglyphs, is abundant in archaeological sites located in the region (See Almeida^8^, Santos Júnior^12^ and De Queirós^13^). Namely, some examples are the municipalities of Antônio Martins (Fig. 11), Timbaúba dos Batistas, Serra Negra do Norte, Caicó, and Jucurutu in the state of Rio Grande do Norte, as well as in Catolé do Rocha, Taperoá, São José das Espinharas, Belém do Brejo do Cruz, São José do Brejo do Cruz, and São Mamede in Paraíba (Fig. 12). This enumeration is merely representative, as the actual number of sites of this typology is considerably larger^12^.

This compilation suggests that indeed, the primary area of occupation of this social group encompasses the central region of the states of Rio Grande do Norte and Paraíba (also known in Brazil as Seridó) although sites with notable similarities can be found at more significant distances, such as in the states of Ceará^13^ and Pernambuco, to the west and south, respectively. In this context, the Serrote do Letreiro Site is identified as situated on the southwest periphery of the territory occupied by this population in the past (Fig. 12). This group left abundant petroglyphs characterized by a distinctive visual language now interpreted as "geometric forms" and an inclination towards *horror vacui*. Throughout this territory, this social group expressed itself graphically in a rather cohesive manner, employing a unique approach to producing petroglyphs and demonstrating a strong preference for specific rock surfaces near water sources for their execution. It is crucial to highlight that the variations observed in the execution techniques and stylistic aspects among the petroglyphs identified at Serrote do Letreiro should not be interpreted as evidence of distinct social authorship, that is, different human cultures. On the contrary, we argue that these variations are better understood as manifestations of individual differences among authors belonging to the same social group. These individuals, while sharing a common graphic or visual identity, express themselves through distinct personal styles.

**Figure 13:**
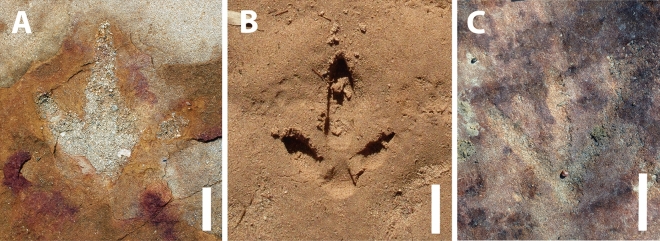
Comparison between tridactyl tracks and tridigit petroglyph. (**A**) Theropod fossil track from Serrote do Letreiro site; (**B**) Recent Palaeognathae track (by Steve Slocomb available on Flickr under CC BY 2.0 Deed); (**C**) Tridigit petroglyph from Serrote do Letreiro site. Scale bar = 5 cm.

Establishing a date for the creation of these petroglyphs poses a significant challenge, not unlike the dating difficulty encountered throughout this territory at other sites, given that there have been very few attempts at dating sites in this region. Human burials of approximately 10,000 years BP were found at Mirador de Parelhas and Pedra do Alexandre, inside what the authors consider the sphere of occupation of the population that crafted the archaeological record found at Serrote do Letreiro and other sites. In Pedra do Alexandre specifically, twenty-eight burials have been dated using radiocarbon, spanning a period from 9400 to 2620 years BP^14,15^. Further research utilizing new methods of direct dating of petroglyphs, such as X-ray fluorescence spectrometry^16^, will certainly shed light on the chronology issue. In the absence of applying absolute dating methods to the petroglyphs, the proposed datings here remain restricted to iconographic inferences, as well as extrapolation from the temporal horizons identified in the few dated sites in the region. Observing such intentionality in the creation of the petroglyphs raises the question of recognition and interpretation of the footprints by the creators of the symbols. The hypothesis that the makers recognized the footprints as such persists even considering that the contemporary understanding of fossils and their association with dinosaurs was likely unknown to the people who first encountered these footprints. It is plausible to argue that, despite the absence of knowledge regarding dinosaurs as we understand them today, the footprints were most likely identified as such due to their formal similarity to rhea footprints (*Rhea americana*—Palaeognathae) (Fig. 13), the largest bird in Brazil, modern theropod dinosaurs, which currently inhabit the Paraíba region. This is supported by the fact that in the same context of the Dinosaur Valley (Sousa, Paraíba), the most renowned fossil trackway is popularly known as the "Rhea's Trail," even though the current population knows of the existence and morphology of dinosaurs.

Regarding the petroglyphs associated with sauropod footprints, their intentionality remains perplexing, given the absence of animals in modern Brazilian fauna whose footprints resemble sauropod footprints. New research, however, may shed light on this. In the municipality of Sousa, a specimen of the proboscidean *Notiomastodon platensis* was dated using radiocarbon in bioapatite corrected to collagen to approximately 24,000 years BP^17^. In the Ceará State, another specimen of *Notiomastodon platensis* was dated using radiocarbon in bioapatite to 7836 years BP^18^. Like sauropods, proboscideans display column-like legs, a result of adaptive convergence related to their large size, which optimizes weight distribution and allows for more efficient locomotion. This results in similar footprints, typically round. A possible mastodon presence in the region during the Pleistocene-Holocene would represent coexistence between these animals and humans. This might indicate that engravers would have recognized the large, round footprints as such, explaining the conspicuity with which petroglyphs are placed near sauropod tracks. Nevertheless, the still precarious amount of evidence for such a hypothesis should point to a cautious identification of petroglyph placement near sauropod tracks as deliberate, unlike the petroglyphs in evident association with the theropod footprints in the northern outcrop, whose deliberate execution is easily explainable. Still concerning the intentionality in the execution of petroglyphs and the appropriation of this materiality by the culture of the group in question, what appears to be a notable reproduction of theropod footprints in the form of tridigit-type engravings can be observed in one petroglyph (Figs. 7G and 13). The identification of these engravings as tridigits that iconically replicate dinosaur footprints provides additional evidence of the possible cultural assimilation of the fossil record. Other tridigit representations have been described as interpretations or reproductions of dinosaur tracks, especially in cave sites near paleontological sites^7^.

Overall, the systematic examination of interactions between humans and the fossil record, such as fossil discoveries in the pre-Columbian era, is a relatively recent scholarly endeavor. Adrienne Mayor played an important role in highlighting the evolution of this research in two significant publications: "The First Fossil Hunters: Paleontology in Greek and Roman Times" (2000)^19^ and "Fossil Legends of the First Americans" (2013)^20^. As early as 1935, there was recognition that the discipline of paleontology is indebted to Native Americans, considering their relevant discoveries, as described by Edward M. Kindle in his notes in the Journal of Paleontology^21^. Despite this, some prominent paleontologists, such as G. Gaylord Simpson, held the opposite view, exemplifying the paradigm that prevailed for most of that time. According to him, pre-Columbian fossil findings were occasional events and are not to be considered in the history of paleontology. Furthermore, he claimed that Native American reports were untrustworthy, being of little ethnological and no paleontological value (see Simpson^22^, 132). This hegemonic conception disregarded native contributions, arguing that fossil discoveries made by indigenous people were the mere result of chance finds, devoid of any scientific continuity (see page 26 in Mayor)^20^. Nonetheless, today, it is indisputable that Native American thought represents a distinct and valuable form of scientific reflection and inquiry. This knowledge, developed over generations, is often referred to as “Native Science”^23^. It is important to highlight that, despite the differences between the Western Scientific Method and Native Science or Traditional Knowledge, both represent equally valid efforts to grasp, describe, and understand the reality that surrounds humans. The integration of Native science knowledge provides a valuable opportunity for academic exchange while at the same time contributing to the empowerment and inclusion of Native American voices in this sphere.

The site in question not only has relevance for historical correction regarding indigenous knowledge concerning the fossil record but also has considerable potential to contribute to the growing discussion regarding fossils as cultural heritage, globally. In recent years, the debate around paleontological heritage and its interface with cultural heritage has grown significantly. Through large-scale mobilizations with high social engagement, such as the restitution of the *Ubirajara jubatus* fossil (See Cisneros et al., p. 15), there emerges an increasingly popular perception of fossils being culturally significant. Concomitantly, more and more paleontological objects arouse the interest of the general public and the market sees these objects as high-priced commodities^24^. Often, these objects are sourced from countries that were once exploration and exploitation colonies. This relates to the fact that former colonial countries typically have legislation that reinforces the prohibition on the removal and departure from national territory of paleontological objects and other cultural property. Such legal frameworks aim to halt the transit of such objects, many of which were taken to Europe during colonial times^25^. Sites such as Serrote do Letreiro, where the profound relationship between native communities and the fossil record is evident, point to the cultural relevance of these objects. This and other instances hold the potential to provide subsidy for the discussion on the importance of these assets and the need to consider them under the same protection, preservation, and promotion measures that other typologies of heritage currently enjoy.

In the Brazilian context, fossils are recognized by the Federal Constitution of 1988, as stipulated in Article 216, as cultural property belonging to the Union. They ought to be protected through all lawful forms of safeguarding and heritage preservation. Although Brazilian legislation recognizes the significance of fossils as cultural objects, Brazilian paleontology and fossils lack specific and comprehensive legislation that would ensure de facto the protection of these elements, which are simultaneously vulnerable and of great interest. The absence of specific regulations for paleontology and fossils in Brazil contributes to inadequate protection for these valuable records^26^. Likewise, it is noteworthy that even elements such as petroglyphs and footprints firmly embedded in the rock face ongoing threats due to bad weather and precarious preservation conditions. Further, there is the risk of theft of rock art and fossilized footprints, which can be removed in rock blocks, supplying an alternative collectors market. Scenarios of theft and illicit trafficking of cultural property highlight the pressing need for more comprehensive and specific legislation, capable of addressing the particularities and demands related to the protection and preservation of fossils and petroglyphs present at the Serrote do Letreiro or other similar sites. The relationship observed at the site between archaeological and paleontological records is evidence of a symbolic and meaningful adoption of the fossil record by human cultures, forming values referring to the identity and memory of pre-colonial groups in Brazil. This leads to the comprehension that the fossil record of that territory must be subject to special protection and precautionary measures aimed at archaeological, historical, and artistic national heritage, as determined in Ordinance nº 375, 2018, chapter V (On paleontological heritage), which establishes the Tangible Cultural Policy of the National Institute of Historic and Artistic Heritage (IPHAN) in Brazil^27^.

Through this preliminary assessment, critical safeguarding measures stand out, such as the need to implement appropriate signs for visitors, highlighting the location of the site, and instructing tourists on fundamental visitation procedures. Similarly, the feasibility of creating 3D replicas of both footprints and petroglyphs is raised, as a form of recording and safeguarding both records. Furthermore, handrails for movement containment could protect fossils and petroglyphs from trampling by humans and animals, as well as from intentional depredation. In consideration of the topographic characteristics of the site, it is proposed that a structure that allows the drainage or redirection of rainwater that accumulates during the rainy season and runs down the slope be designed, thus mitigating the dragging of debris across the surface of the outcrop.

## Methods

For general reconnaissance, a pedestrian survey oriented in a north–south direction, and a repeated survey in the opposite direction were undertaken twice in the spring of 2023. These field surveys were specifically aimed at identifying new fossilized footprints and petroglyphs, expanding the search past the previously known findings made between 1975 and 2022. No subsurface testing was conducted during these surveys, focusing entirely on surface observations. An aerial and space-based survey was implemented to generate aerial photographs and identify potential new rock outcrops that could be concealed by vegetation. Such photogrammetry is used to obtain reliable metric information regarding physical objects and the environment, in this instance through the use of a drone. It has been widely employed in cartographic production and the imagistic study of sites in natural and cultural heritage for several years (Camargo Tuta^28^; ASPRS apud Limaverde^29^, p. 207). Photogrammetry’s aim in archaeological and paleontological research is the digital preservation of heritage sites, leveraging technological resources to capture reality. Through photogrammetry, it is possible to carry out high-precision documentation, aiming to conserve data and provide realistic visualizations of the object. The quality of the documentation significantly impacts the extent of analysis and the depth of results. In this sense, the more information the photographic record can provide, the better the understanding of the inherent phenomena in the archaeological and paleontological record^30^. Furthermore, photogrammetry allows researchers to characterize, identify, and enhance the visual information of motifs through the manipulation of photographic contrast tools, and tracing, as well as proposing possible relationships between them through free joining and overlapping. The technique is considered indispensable to 'freeze in time' visually imperceptible data, especially in the face of risks and threats posed by agents of natural degradation affecting the site’s preservation and readability. The photogrammetry method used in this study follows the guidelines proposed by Mark & Billo^31^, Munõz & Trujillo^32^, and Pereira et al.^33^, in addition to the procedures adopted by Pessis^34^ for the photographic survey of the fossiliferous record and rock art distributed on horizontal panels.

The Drone Phantom 4 prev V2 with an image georeferencing system and a Canon EOS 5D Mark III camera with a 24–105 mm Ultrasonic macro lens were chosen to dimensionalize and analyze fossil and pictorial records. The first device features a 1'' CMOS sensor capable of producing 4 K resolution images, and the second provides a total framing of 22.3 MP. In the process of capturing the observed reality, following Pessis' guidelines, the strategy was to document the entirety of the rock outcrops and the fossil and pictorial space. This involved segregating the outcrops into study panels, arbitrarily defined to respect the maximum visibility limit of the motifs without compromising the quality and resolution of the results obtained with the drone, or risking safe zoom usage during the digital manipulation of the photographic content in the course of the analysis stage. The flight programming that included these two surveys was carried out at a focal distance of 1.80 m and 30 m perpendicular to the planes with an overlap of the established shots at 90%. Therefore, 53 images were acquired from the first outcrop and 37 from the second. The individual capture of petroglyphs and footprints was executed with the manual camera, following the minimum requirement of 3 photos per unit, generating 357 images using scales of 10 cm and 5 cm (3.94 and 1.97 inches). The geoprocessing of the images used to create the mosaics here analyzed was carried out using the PIX4DMapper software. Tracings of the petroglyphs were digitally created in Adobe Illustrator version 27.0.1 X64 and rendered vectorially after image post-processing. These adjustments included increased contrast and sharpness, aiming to enhance the visibility of the details in the petroglyphs. A collection of petroglyphs and tracks was digitally compiled to improve comprehension of the relationship between these records (Supplementary Information).

Social Inclusive Archaeology, a branch of Public Archaeology, was employed as a theoretical-methodological framework. Social inclusive archaeology is defined as an approach that aims for social transformation, focusing on youth protagonism. It is characterized as a process of inclusion since childhood, positioning the community's youth as guardians of local memory, building citizenship and dignification^29^. Thus, two pre-adolescent students accompanied the fieldwork. They are members of the Casa Grande Foundation—Kariri Man Memorial, a non-profit organization that aims to offer the youth of the Ceará backcountry vocational training and learning in various areas such as theater, photography, museology, art curation, history, and archaeology. In this sense, the involvement of pre-adolescents in fieldwork resulted in the acquisition of experience and immersion in archaeological and paleontological field research procedures and constitutes an action of heritage education and engagement in scientific literacy. The participation of these young individuals resulted in a report to the Casa Grande Foundation, summarizing the learnings from the experience and consolidating the knowledge and benefits resulting from applying theoretical-methodological approaches that encompass the local population and youth.

### Supplementary Information

Supplementary Information.

## Data Availability

The data that support the findings of this study are available from the authors on reasonable request, see author contributions for specific contact information.
