# Author Correction: A remarkable assemblage of petroglyphs and dinosaur footprints in Northeast Brazil

**DOI:** 10.1038/s41598-024-65283-y

**Published:** 2024-06-21

**Authors:** Leonardo P. Troiano, Heloísa B. dos Santos, Tito Aureliano, Aline M. Ghilardi

**Affiliations:** 1 The National Historic and Artistic Heritage Institute, Brasília, DF 70390-135 Brazil; 2 Institute of Cariri Archaeology Dr. Rosiane Limaverde, Casa Grande Foundation, Nova Olinda, CE 63165-000 Brazil; 3 Department of Zoology, Regional University of Cariri (URCA), Crato, CE 63105-000 Brazil; 4 Diversity, Ichnology and Osteohistology Laboratory (DINOLab), Department of Geology, Federal University of Rio Grande do Norte (UFRN), Natal, RN 59075-000 Brazil

Correction to: *Scientific Reports* 10.1038/s41598-024-56479-3, published online 19 March 2024

The Acknowledgements section in the original version of this Article was incomplete.

“We are grateful to Arthur Ferreira Sampaio for his invaluable assistance in capturing the drone images, and to Renan Rodrigues Chandu and Pedro Arcanjo José Feitosa, and the Casa Grande boys for their contributions in capturing petroglyph and site photographs, as well as assisting in the compilation of field notes. We appreciate Dr. Adrienne Mayor’s expertise and insights into the archaeological investigation of human-fossil interactions. We also would like to thank Zarah Trindade Gomes, Rebecca Erickson, and Francisco Tibério Felizmino de Araújo for their indispensable help in collecting field data; the family of Mr. Chico de Luca for always welcoming us to their farm and taking the best possible care of the Letreiro site. Funding for the authors was provided by the Foundation for Support to Scientific and Technological Development of Ceará (FUNCAP 09/2023) (T.A.) and the Federal University of Rio Grande do Norte (UFRN PIB21712-2023) (A.M.G.).”

now reads:

“We are grateful to Arthur Ferreira Sampaio for his invaluable assistance in capturing the drone images, and to Renan Rodrigues Chandu and Pedro Arcanjo José Feitosa, and the Casa Grande boys for their contributions in capturing petroglyph and site photographs, as well as assisting in the compilation of field notes. We appreciate Dr. Adrienne Mayor’s expertise and insights into the archaeological investigation of human-fossil interactions. We also would like to thank Zarah Trindade Gomes, Rebecca Erickson, and Francisco Tibério Felizmino de Araújo for their indispensable help in collecting field data; the family of Mr. Chico de Luca for always welcoming us to their farm and taking the best possible care of the Letreiro site. Funding for the authors was provided by the Foundation for Support to Scientific and Technological Development of Ceará (FUNCAP 09/2023) (T.A.) and the Federal University of Rio Grande do Norte (UFRN PIB21712-2023) (A.M.G.). This study was financed in part by the Coordenação de Aperfeiçoamento de Pessoal de Nível Superior - Brasil (CAPES) - Finance Code 001.”

The original Article has been corrected.

